# Predictors of sickness absence among employees with common mental disorders in Sweden– a longitudinal study

**DOI:** 10.1186/s12889-025-21563-4

**Published:** 2025-02-03

**Authors:** Anna Frantz, Anna Toropova, Iben Axén, Gunnar Bergström, Anna Finnes, Elisabeth Björk Brämberg

**Affiliations:** 1 Unit of Intervention and Implementation Research for Worker Health, Institute of Environmental Medicine, Karolinska Institutet, Box 210, Stockholm, 171 77 Sweden; 2 Department of Occupational Health, Psychology and Sports Sciences, Faculty of Health and Occupational Studies, University of Gävle, Gävle, Sweden; 3 Division of Insurance Medicine, Department of Clinical Neuroscience, Karolinska Institutet, Stockholm, Sweden; 4 Academic Primary Healthcare Centre, Region Stockholm, Stockholm, Sweden; 5 School of Public Health and Community Medicine, Institute of Medicine, University of Gothenburg, Gothenburg, Sweden

**Keywords:** Worker, Mental health, Return to work, RTW, Occupational health

## Abstract

**Background:**

The study aimed to explore which sociodemographic, health-related, and work-related factors were associated with the number of sickness absence days during 18 months among employees on sickness absence due to common mental disorders.

**Methods:**

A longitudinal study with participants from a cluster-randomized controlled trial was conducted. Participants from both intervention and control groups were treated as one cohort. Factors included in the analysis were sociodemographic, health-related, and work-related variables collected through questionnaires at baseline. The outcome was cumulative net sickness absence days for sickness absence spells exceeding 14 days and was collected from a national register. Data was analyzed using generalized estimating equations.

**Results:**

The sample consisted of 197 employees. Lower-rated work ability in relation to physical demands ([exp (B) 1.19], 95% CI 1.02–1.40) and higher-rated job demands ([exp (B) 1.28], 95% CI 1.01–1.61), were associated with increased number of sickness absence days during the 18 months follow-up. Higher certainty of return to work within three months ([exp (B) 0.63], 95% CI 0.48–0.83) was associated with a decreased number of sickness absence days during the 18 months follow-up.

**Conclusions:**

Our study suggests that work-related factors, i.e., high job demands and impaired work ability, are associated with an increased number of days on sickness absence. Additionally, the certainty of returning to work within three months is associated with fewer days on sickness absence. The results highlight the importance of addressing specific workplace factors when designing interventions aimed at decreasing sickness absence for employees on sickness absence due to CMDs. The results could be used to inform a dialogue between healthcare personnel and employees on sickness absence due to CMDs, and to serve as basis for a structured inventory to assist healthcare personnel in addressing workplace factors.

## Background

Common mental disorders (CMDs), i.e. anxiety, depression, and stress-related disorders, affect nearly one in five workers yearly around the globe [1, 2], and depression stands out as a major global cause of disability [3]. Having a CMD impacts functioning and leads to individual suffering in terms of emotional distress, risk of social isolation, and threat to economic income [4, 5]. For organizations and societies, CMDs pose a challenge in terms of production loss and societal costs due to sickness absence [6].

Approximately 30% of employees with CMDs across OECD countries experience a sickness absence episode during their lifetime [7]. Sickness absence due to CMDs has a high rate of recurrence, with approximately 20% of affected individuals experiencing a new episode within a year from the first occurrence [8]. Moreover, sickness absence due to a CMD increases the risk of becoming excluded from the labor market through disability pension and unemployment. In Sweden, mental disorders, with CMDs being the largest diagnosis group, represent 50% of sickness absence for women and 39% for men [9].

Interventions aiming at reducing sickness absence among employees with CMDs can be roughly divided into clinical interventions, work-directed interventions, or a combination of the two [10–13]. Clinical interventions seek to reduce symptoms, enhance coping strategies related to work [10, 11, 13], and/or introduce activities facilitating recovery, such as mindfulness or yoga [10]. Work-directed interventions focus on modifying the work environment and introducing a graded exposure to work [11]. Although some clinical interventions, such as psychological interventions, and a combination of clinical and work-directed interventions may have a small effect on sickness absence days, high-quality studies are still needed to identify what interventions are effective and for whom [10–13]. Exploring predictive factors of sickness absence in a clinical context is important for the development of effective interventions and the identification of groups that may benefit from these interventions [14]. Primary healthcare is the first-line psychiatry in Sweden and is where the major proportion of adults with CMDs receive care [15]. Besides the treatment of symptoms of CMDs, general practitioners certify sickness absence when needed [16, 17] and refer the patient to further rehabilitation. There is therefore a need to further explore factors associated with sickness absence for employees with CMDs in primary healthcare [10, 11].

Predictive factors have been identified for entering sickness absence among working populations [18], return to work (RTW) for employees on sickness absence [19], or a combination of these [20]. Female gender, higher symptom severity/stress levels, and poor general health are associated with increased risk of sickness absence due to CMDs [19–21]. High job demands, low job control, and job strain are also associated with sickness absence [18, 20, 22], as well as working in healthcare, education, and social service sectors [21, 23]. Social support, positive expectations about the RTW process, and RTW self-efficacy are associated with earlier RTW [19, 20].

Studies on predictive factors for sickness absence due to CMDs have increased in numbers [20]. Although factors linked to the individual and work have been identified, de Vries et al. [20] still highlight the need for further research on work-related factors, such as decision latitude, job sector, and overtime work. Longitudinal studies, using register data as an outcome measure, can provide additional knowledge in this area. To our knowledge, factors associated with sickness absence among a population of employees on sickness absence due to CMDs in primary healthcare have not yet been explored.

The aim of this study was to explore which sociodemographic, health-related, and work-related factors were associated with the number of sickness absence days during 18 months among employees on sickness absence due to common mental disorders.

## Method

### Study design

This longitudinal study followed participants from a cluster-randomized controlled trial (RCT) in Swedish primary healthcare (ClinicalTrials.gov registration identifier: NCT03346395, registered January 12, 2018) [24]. In the present study, participants from the cluster-RCT were treated as one cohort, regardless of allocation.

### Setting

The study was set in primary healthcare in western Sweden, at 24 primary care centers (PCCs). The PCCs were publicly funded and located in both rural and urban areas.

### Study participants

The inclusion criteria were age between 18 and 59 years, current sickness absence from work due to CMDs for 2–12 weeks issued by a physician at participating PCCs, and acceptance of employer involvement in the intervention. Exclusion criteria were pregnancy, a severe mental disorder or co-morbidity considerably affecting work ability, or being subjected to workplace bullying.

### Recruitment process

Patients eligible for recruitment were identified in PCC registers and received written information about the study. Participants signed informed consent before inclusion. For a more detailed description of the recruitment procedure, see Björk Brämberg et al. [24]. Recruitment began in February 2018 and ended in February 2020. To account for the possibility that sickness absence days may have been affected by the COVID-19 pandemic, a control variable was added indicating if the participant’s follow-up period coincided with the COVID-19 pandemic (yes/no).

### Data collection

Register data on sickness absence 24 months before and 18 months after baseline was retrieved from the Swedish Social Insurance Agency’s registry Micro Data for the Analyses of Social Insurance. Sickness absence pay during the first 14 days is covered by the employer in Sweden. From day 15, sickness absence pay is administered by the Swedish Social Insurance Agency. Therefore, register data on sickness absence is only available for episodes lasting 15 days or more. For the exposure variables, data was collected through web-administered questionnaires upon inclusion.

### Outcome

**Sickness absence days** for the 18-month study period were summed up into net sickness absence days (i.e., two days of sickness absence at 50% is equal to 1 day net sickness absence) and rounded to integers. In Sweden, sickness absence can be certified for 25, 50, 75 or 100% of the full working day.

### Exposure variables

#### Sociodemographic variables

Information on age, gender, educational level, cohabiting, children living at home, and country of birth was collected. Country of birth was not included in the analysis due to a small variance (only 7% of the study sample was born outside Sweden).

#### Health-related variables

Self-reported symptoms of anxiety and depression were measured with the Swedish version of the Hospital Anxiety and Depression Scale (HAD) [25]. The scale consists of 14 items representing two subscales: depression (7 items) and anxiety (7 items) [26]. Each item is graded on a 4-point Likert scale. For each subscale, the score is the sum of the 7 items respectively (range 0–21), where a higher sum represents more severe symptoms. HAD is a valid measure for assessing symptoms and identifying cases of depression and anxiety in a primary healthcare population [27]. The Cronbach’s α in the current sample was 0.85 for the anxiety subscale and 0.86 for the depression subscale. Stress-related exhaustion was assessed using the questionnaire Self-rated Exhaustion Disorder (s-ED) [28]. The s-ED questionnaire comprises four items capturing experiences of exhaustion, strain over time, and specific symptoms related to exhaustion disorder, classified on a three-point ordinal scale: no, light/moderate, or pronounced ED. The first three items have a yes/no response option. The third item lists six common symptoms of ED, while the fourth item provides a differentiation between light/moderate and pronounced ED. The scale has been tested for construct validity and found to correspond well to other constructs of mental health, as well as predicting future sickness absence [28].

Sleeping problems were assessed with four items on sleep quality from the Karolinska sleep questionnaire (KSQ) [29], e.g., “Difficulties falling asleep” and “Repeated awakenings with difficulties falling asleep again”. Response alternatives were 0– never, 1– seldom (occasionally), 2– sometimes (several times per month), 3– often (1–2 times per week), 4– most of the times (3–4 times per week), or 5– always (5 times or more per week). The mean score for the items was calculated in accordance with previous literature, where 0 is best and 5 is worst [29]. The Cronbach’s α for the sample was 0.82. Quality of life was assessed with the European quality of life– five dimensions (EQ-5D) questionnaire [30]. The EQ-5D questionnaire is a generic instrument covering five items concerning mobility, personal hygiene, daily activities, pain, and anxiety/depression answered on a 3-point scale, where 1 is best and 3 is worst [30]. The index ranged from 0 (worst) to 1000 (best). The EQ-5D is a valid measure for patients with depression and to some extent anxiety [31]. General health was assessed with one item from the Shortform-36 (SF-36) [32, 33] The item was “In general, would you say your health is?”, answered on a 5-point scale (1– Excellent, 2– Very good, 3– Good, 4– Fair, and 5– Poor) [33].

#### Work-related variables

Data on the following variables concerning the person’s work situation were collected: employment conditions (0– permanent, 1– temporary), managerial position (0– no, 1– yes), overtime work (estimated on a 6-point scale ranging from 1– every day to 6– never), and work sector (0– public, 1– private).

Work ability was measured using three items from the Work Ability Index (WAI) [34] concerning the work ability in relation to mental (e.g. knowledge, creativity, responsibility, and cooperation ranging from 1– excellent to 5– very bad) and physical (e.g. strength, perseverance, mobility, and dexterity ranging from 1– excellent to 5– very bad) workload, and one’s prognosis of work ability for the next two years (ranging from 1– unlikely to 3– relatively certain). One item concerning one’s prediction of RTW was added, namely *How certain are you that you will be back in your current job during regular working hours in three months?* The question was answered on a 3-point scale (1– Probably not, 2– I’m not sure about that, 3– I’m pretty sure). This type of single-item question has proven to be a valid measure compared with multi-item measures when predicting work-related outcomes [35].

To assess emotional demands at work, four items from the Swedish version of the Copenhagen Psychosocial Questionnaire (COPSOQ v II) [36] were used, e.g., “Is your work emotionally demanding?”. The items were rated from 1 to 5, where 1 represents low emotional demands. An index was computed if participants had answered at least two of the four items. COPSOQ has been found a valid measure [36]. Cronbach’s α for this sample was 0.87. Five items from the General Nordic Questionnaire for psychological and social factors at work (QPSNordic) [37] were used: conflict with values “Does your work include tasks that conflict with your personal values?”; fair leadership “Does your immediate superior treat the workers fairly and impartially?”; reward “At your organization, are you rewarded (money, encouragement) for a job well done?”; work-to-home interference; and home-to-work interference. The items were answered on a 5-point scale ranging from 1– very seldom or never to 5– always. The QPSNordic is a reliable instrument with predictive validity for long-term sickness absence (> 90 days) [38]. Although the use of single items may affect the validity of the responses, the questions were straightforward and chosen to capture the essence of the underlying construct without over-burdening the respondents. The Demand-control-support questionnaire (DCSQ) was used providing assessment of psychological demands (e.g. speed, intensity, conflicting demands), decision latitude (e.g. skill, creativity, influence on how work is carried out), and social support (e.g. support from colleagues, getting along with supervisor, atmosphere at workplace) [39] with corresponding Cronbach’s α: (0.82), (0.62), and (0.82). Items are graded on a 4-point Likert scale ranging from 1– often to 4– never/almost never for the first two dimensions, and 1– strongly agree to 4– strongly disagree for the third dimension. The DCSQ has shown satisfactory psychometric properties both among a general population and among a population with depression [39].

### Confounders and bias

Group allocation, diagnosis at baseline, follow-up during COVID-19, sickness absence days 24 months before baseline, and tenure were considered potential confounders and adjusted for in the final model. To minimize the risk of information bias, the outcome sickness absence was obtained from a register, and validated measures on health- and work-related factors were used.

### Statistical analysis

Mean, standard deviation, and Cronbach´s α were computed for indexes. Moreover, counts and percentages were used to present categorical and ordinal variables. Spearman’s correlation coefficient was computed to check for multicollinearity, with values over 0.5 considered problematic and therefore were further examined [40]. The variables DCSQ psychological demands and work-home interference had a correlation coefficient of 0.53. Neither of the variables were highly correlated to other variables and thus were kept in the analysis (Table 1). In addition, multicollinearity was assessed by computing the variance inflation factor (VIF) and tolerance statistics. Although there is no established cut-off, a value above 10 and an average VIF substantially greater than 1 was considered problematic [40]. The VIF ranged from 1.2 to 2.1 for the independent variables, and the tolerance level was between 0.4 and 0.8. The VIF was well below 10, and the average VIF was close to 1, with a tolerance level above 0.01, suggesting a low risk of multicollinearity [40]. Scatterplots were visually assessed for a linear relationship between the variables and the outcome.

**Table 1:** Participant characteristics at baseline of the study sample (*n* = 197) and cumulative sickness absence days during 18-months follow-up

Factors	*n* (%)	Mean (SD)	Median (IQR)
Sociodemographic			
**Gender**, woman	167 (85)		
**Age**		42 (10.3)	
**Born outside Sweden**, yes	13 (7)		
**Cohabiting**, yes	133 (68)		
**Children under 16 living at home**, yes	103 (52)		
**Educational level**			
Compulsory school	7 (4)		
Secondary school	94 (48)		
University or equivalent	81 (41)		
**Health-related**			
**HAD Anxiety**		11.2 (4.43)	
**HAD Depression**		9.9 (4.14)	
**s-ED**			
No	23 (12)		
Light/moderate	27 (14)		
Pronounced	129 (65)		
**Emotional demands**, COPSOQ		2.6 (1.02)	
**Sleep quality index**, KSQ insomnia subscale		3.4 (1.28)	
**SF-36 general**		3.7 (1.03)	
Excellent/very good	27 (14)		
Good	40 (20)		
Fair/Poor	116 (59)		
**EQ-5D-index**		597 (235.7)	
**Work-related**			
**Permanent employment**	170 (86)		
**Managerial position**	24 (12)		
**Overtime work**		4.1 (1.57)	
**Work sector**			
Public	107 (54)		
Private	66 (34)		
**Work ability - Physical demands**, WAI		2.8 (1.20)	
**Work ability– Mental demands**, WAI		3.7 (0.97)	
**Prognosis of work ability in the next 2 years**, WAI			
Probably not	30 (15)		
I am not certain of it	59 (30)		
I think so	91 (46)		
**Certainty of RTW < 3 months**			
Probably not	15 (7		
I am not certain of it	77 (39)		
I think so	88 (45)		
**Psychological demands**, DCSQ		3.0 (0.65)	
**Social support**, DCSQ		2.9 (0.57)	
**Decision latitude**, DCSQ		2.9 (0.45)	
**Conflict with values**, QPS Nordic		2.4 (1.12)	
Never/almost never/seldom	91 (46)		
Sometimes	61 (31)		
Often/very often/always	28 (14)		
**Fair leadership**, QPS Nordic Never/almost never/seldom	38 (20)	3.6 (1.24)	
Sometimes	36 (18)		
Often/very often/always	104 (52)		
**Reward**, QPS Nordic		2.2 (1.12)	
Never/almost never/seldom	105 (54)		
Sometimes	52 (26)		
Often/very often/always	22 (10)		
**Work-to-home interference**, QPS Nordic		3.5 (0.98)	
Never/almost never/seldom	23 (11)		
Sometimes	57 (29)		
Often/very often/always	103 (53)		
**Home-to-work interference**, QPS Nordic		2.5 (1.2)	
Never/almost never/seldom	96 (49)		
Sometimes	43 (22)		
Often/very often/always	44 (23)		
**Confounders**			
**SA days 24 months before baseline**		51 (67.8)	23 (11–57)
**Primary SA diagnosis at inclusion**			
Depression	48 (24)		
Anxiety	46 (23)		
Adjustment disorder	103 (53)		
**Follow-up during covid**, yes	73 (37)		
**Work experience** Less than one year (1)	30 (15)		3 (2–4)
1–2 years	41 (21)		
3–5 years	43 (22)		
6–10 years	26 (13)		
More than 10 years (5)	42 (21)		
**Main outcome**			
Sickness absence days			67 (18–175)

Disperse numbers are due to missing data

A general estimating equation (GEE) analysis was adopted to explore the relationship between the exposure factors and sickness absence with a negative binomial distribution and log link function. The GEE analysis has the advantage of handling count data, skewed distribution of the outcome sickness absence days, and the possible dependence between observations in the clusters [41]. An independent correlation matrix was chosen. Missing data was not imputed and ordinal variables were treated as continuous to minimize loss of power in the analysis [42].

The GEE analysis was performed in two steps. In step one, univariable modeling was conducted for all independent variables. In step two, all variables that generated a p-value of *p* ≤ 0.20 were added hierarchically to a multivariable model in blocks of sociodemographic (block 1), health-related (block 2) and work-related (block 3) variables. The p-value 0.20 was chosen not to omit any variable that could be associated with the outcome, but also to avoid overfitting the final model, and has been used in previous research [43]. Backward selection was applied to decide which variables to keep in the final model, as it is preferable for exploratory model building [40]. Variables with the highest p-value were removed until only variables with *p* < 0.05 remained in the models. If the quality under the information criterion (QIC) changed by more than 10%, the variable was re-entered into the model and the variable with the second highest p-value was removed. All data analysis was conducted in IBM SPSS Statistics version 28.

### Ethical considerations

Ethical approval was obtained for the original cluster-RCT at the local Review Board in Gothenburg (nr 496 − 17). Additional ethical approval for the research question in the study at hand was obtained from the Swedish Ethical Review Authority (nr 2021/01768).

## Results

### Participants

**Fig. 1:**
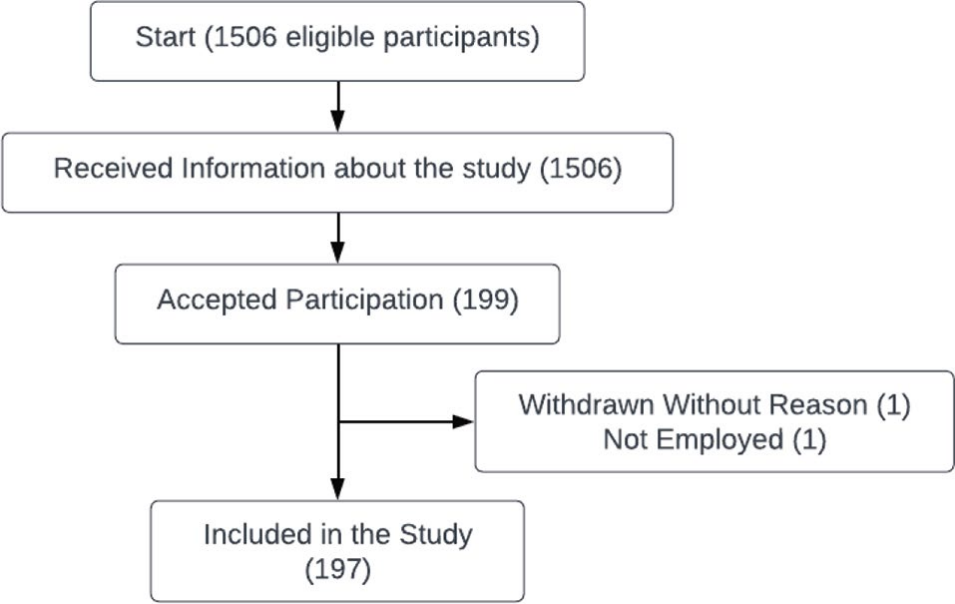
Flowchart of the study

A total of 1506 eligible patients received information about the original cluster RCT, whereof 74% were women and mean age of 39 years. Of these, 199 accepted to participate. Two participants were excluded after allocation. Remaining for inclusion were 197 participants (Fig. 1). A total of 12 participants did not respond to the baseline questionnaire. Among the non-responders, a greater proportion were male (25% vs. 15%) and had their follow-up period during the COVID-19 pandemic (50% vs. 36%).

### Descriptives

Participant characteristics are presented in Table 2. For the baseline variables, missing values ranged between 6 and 10%. The participants were mainly women and born in Sweden. The mean age was 42 years. The mean ratings of anxiety and depression were above the suggested clinically relevant cut-off of eight [27], and 65% reported symptoms of pronounced exhaustion disorder. The median days of sickness absence during the 18-month study period for the total sample was 67, with an interquartile range of 18–175 days.

**Table 2:** Correlation matrix

	1	2	3	4	5	6	7	8	9	10	11	12	13	14	15	16	17	18	19	20	21	22	23	24	25	26	27	28
1. Gender	1																											
2. Age	0.14*	1																										
3. Cohabiting	0.02	0.11	1																									
4. Children living at home	-0.02	0.06	-0.01	1																								
5. Education	-0.15*	0.02	0.00	0.01	1																							
6. HAD anxiety	0.01	-0.21**	0.05	-0.01	-0.18*	1																						
7. HAD depression	0.10	-0.10	-0.08	-0.03	-0.15*	0.46**	1																					
8. s-ED	0.03	0.03	0.08	0.10	-0.03	0.29**	0.37**	1																				
9. COPSOQ Emotional demands	0.12	0.04	0.06	-0.02	-0.17*	-0.10	-0.12	-0.12	1																			
10. KSQ sleep quality	0.08	0.01	-0.14	0.05	-0.02	0.19*	0.21**	0.09	-0.04	1																		
11. SF-36 single item	-0.05	0.01	0.01	-0.02	-0.14	0.29**	0.33**	0.22**	-0.02	0.12	1																	
12. EQ-5D	-0.06	-0.04	0.07	-0.05	0.07	-0.42**	-0.35**	-0.35**	0.17*	-0.21**	-0.45**	1																
13. Employment conditions	-0.08	0.03	-0.02	-0.04	0.10	0.08	0.11	0.05	0.02	-0.01	0.05	-0.14	1															
14. Managerial position	0.17	-0.21**	-0.09	-0.11	-0.14	0.12	0.07	-0.03	0.07	0.01	-0.05	0.06	-0.03	1														
15. Overtime work	-0.04	0.16*	0.12	0.13	-0.10	-0.08	-0.09	-0.07	0.04	-0.50	0.06	0.13	-0.05	-0.16*	1													
16. Work sector	0.27**	-0.19*	0.05	0.04	-0.06	0.07	-0.02	0.07	0.34**	0.15	-0.03	-0.01	-0.09	0.22**	-0.11	1												
17. Work ability physical demands	0.08	0.13	-0.04	-0.07	-0.14	0.14	0.15*	0.23**	0.04	0.18*	0.41**	-0.34**	-0.03	0.15*	-0.11	0.08	1											
18. Work ability mental demands	0.17	-0.08	-0.10	0.04	0.01	0.25**	0.30**	0.38**	-0.18*	0.11	0.35**	-0.42**	0.13	0.09	-0.08	0.16*	0.41**	1										
19. Work ability 2 years	-0.13	0.02	0.08	0.05	0.21**	-0.18*	-0.21**	-0.22**	0.12	-0.23**	-0.12	0.11	0.03	-0.29**	0.08	-0.11	-0.34**	-0.21**	1									
20. Certainty of RTW < 3 months	-0.13	0.04	0.04	-0.04	-0.02	-0.28**	-0.26**	-0.28**	0.13	-0.27**	-0.31**	0.41**	-0.04	-0.11	0.08	-0.12	-0.31**	-0.47**	0.30**	1								
21. DCSQ demands	0.01	-0.09	-0.07	0.03	0.05	0.13	0.15*	0.24**	-0.27**	0.13	0.15*	-0.17*	0.13	0.06	-0.37**	0.02	0.20**	0.28**	-0.26**	-0.30**	1							
22. DCSQ support	-0.05	0.08	0.09	-0.01	0.10	-0.13	-0.16*	-0.11	0.26**	-0.12	-0.15*	0.16*	0.01	-0.12	0.22**	-0.04	-0.29**	-0.16*	0.39**	0.26**	-0.42**	1						
23. DCSQ decision latitude	-0.02	0.08	-0.02	0.11	0.39**	0.02	-0.05	0.04	-0.21**	-0.03	0.02	-0.15*	-0.02	-0.11	-0.08	-0.04	-0.16*	0.06	0.25**	-0.05	0.06	0.19*	1					
24. QPS Nordic conflict values	-0.04	0.01	-0.05	0.01	0.06	0.22**	0.09	0.05	-0.36**	0.09	0.04	-0.15*	0.11	0.15*	-0.11	-0.19*	0.16*	0.11	-0.17*	-0.19*	0.29**	-0.37**	-0.02	1				
25. QPS Nordic fair leadership	0.01	0.05	0.05	-0.05	0.02	-0.05	-0.14	-0.05	0.16*	-0.06	-0.11	0.06	0.02	-0.06	0.10	-0.01	-0.17*	-0.16*	0.31**	0.15*	-0.29**	0.42**	0.09	-0.31**	1			
26. QPS Nordic reward	0.14	-0.08	0.10	-0.02	0.02	0.10	-0.19*	-0.11	0.14	-0.02	-0.15*	0.16*	-0.11	0.11	0.08	0.18*	-0.22**	-0.11	0.21**	0.08**	-0.26**	0.41**	0.08	-0.20**	0.31	1		
27. Work-home interferance	0.03	-0.08	-0.08	0.08	0.08	0.15*	0.18*	0.28**	-0.24**	0.23	0.07	-0.17*	0.07	0.01	-0.21**	-0.02	0.13	0.26**	-0.27**	-0.28	0.53**	-0.30**	0.11	0.12	-0.21**	-0.17*	1	
28. Home-work interference	-0.12	-0.11	-0.06	0.36**	0.14	0.03	0.12	0.19*	-0.12	0.03	0.13	-0.06	-0.05	-0.05	0.09	-0.07	0.03	0.07	-0.01	-0.13	-0.04	0.13	0.14	-0.03	-0.09	-0.03	0.08	1

*p < 0.05, **p < 0.001

### Main results

From the univariable GEE analysis, one sociodemographic, four health-related and ten work-related variables had a p-value of ≤ 0.20, resulting in 15 variables included for further multivariable analysis (Table 3). Variables not included in the multivariable analysis were gender, educational level, cohabiting, children living at home, HAD anxiety, s-ED, emotional demands, managerial position, work ability 2 years, DCSQ decision latitude, QPS Nordic conflicting values, QPS Nordic fair leadership, and home-work interference.

**Table 3:** Variables with p-value ≤ 0.20 from univariable GEE analysis

Factor	B	CI	*p*-value
Sociodemographic			
Age	0.01	-0.003; 0.028	0.126
Health-related			
HAD depression	0.06	0.023; 0.098	0.001
KSQ sleep quality	0.16	0.046; 0.277	0.006
SF-36 single item	0.23	-0.003; 0.466	0.053
EQ-5D	-0.00	-0.002; -0.001	< 0.001
Work-related			
Employment conditions	-0.73	-1.762; 0.305	0.167
Overtime work	-0.08	-0.179; 0.010	0.081
Work sector	0.03	-0.074; 0.137	0.093
Work ability physical demands	0.24	0.085; 0.403	0.003
Work ability mental demands	0.34	0.102; 0.570	0.005
Certainty of RTW < 3 months	-0.62	-0.847; -0.393	< 0.001
DCSQ demands	0.33	0.076; 0.681	0.011
DCSQ support	-0.34	-0.635; -0.042	0.017
QPS Nordic reward	-0.15	-0.305; 0.002	0.052
Work-home interference	0.16	0.005; 0.317	0.044

The result from the hierarchical model building with backward selection is presented in Table 4. None of the sociodemographic (block 1) or health-related (block 2) variables remained in the final model. The final model contained three work-related (block 3) variables, work ability in relation to physical demands ([exp (B) 1.19], 95% CI 1.02–1.40), DCSQ psychological demands ([exp (B) 1.28], 95% CI 1.01–1.61), and certainty of RTW within three months ([exp (B) 0.63], 95% CI 0.48–0.83). For a one-unit increase of impaired work ability in relation to physical demands, the rate of sickness absence days increases by 19%. Due to the sample size and the explorative approach of the statistical analysis, the results should be interpreted with caution and calculation of exact days was not possible.

**Table 4:** Results of the hierarchical GEE analysis for socio-demographic, health- and work-related variables on sickness absence

Factor	*N*					Adjusted for confounders		
		Exp (B)		95% CI	QIC	Exp (B)	95% CI	QIC
**Block 1**	197							
Intercept		67.59						
Age		1.01		0.99;1.03				
**Block 2**	174				369.387			
Intercept		113.31						
HAD depression		1.05*		1.01; 1.10				
EQ-5D		0.99*		0.98: 0.99				
SF-36 single item		1.11		0.82: 1.50				
KSQ sleep quality		1.13		0.99; 1.27				
**Block 3**	162				354.848			
Intercept		92.96						
HAD depression		1.03		0.99; 1.07				
EQ-5D		1.00		0.99; 1.00				
Employment conditions		1.41		0.51; 3.90				
Overtime work		0.99		0.89; 1.10				
Work sector		1.09		0.75; 1.10				
Work ability physical demands		1.19*		1.02; 1.40		1.19*	1.02; 1.39	349.786
Work ability mental demands		1.12		0.89; 1.46				
Certainty of RTW < 3 months		0.63**		0.48; 0.83		0.63**	0.49;0.80	
DCSQ demands		1.28*		1.01; 1.61		1.30*	1.03;1.64	
DCSQ support		1.03		0.71; 1.52				
QPS Nordic reward		0.98		0.82; 1.16				
Work-home interference		0.91		0.75; 1.10				

Notes Exp (B) = exponentiated beta coefficients; CI = confidence interval; QIC = Quality under the information criterion

*p < 0.05,**p < 0.001

## Discussion

### Key findings

This study explored which sociodemographic, health-, and work-related factors were associated with the number of sickness absence days during 18 months among employees on sickness absence due to a CMD. Lower ratings of work ability in relation to physical demands and higher ratings of psychological demands (e.g., speed, intensity, conflicting demands) were both associated with more days of sickness absence during the 18-month follow-up. Higher certainty of returning to work at ordinary working hours within three months was associated with fewer days of sickness absence.

### Discussion in relation to previous literature

Lower self-assessed work ability in relation to physical demands was associated with more days on sickness absence during the follow-up. The results of our study suggest a relationship between CMDs, ability to perform physical tasks at work, and sickness absence, supporting the need for exploring activity limitations in relation to sickness absence among employees with CMDs [20]. Approximately 30% (60/197) of our sample worked in healthcare, where physical demands may be high in combination with low control and limited resources, possibly explaining why this variable was important in explaining the number of sickness absence days in our sample. CMDs and physical workload have also been linked to future disability retirement [44], in line with our results. Another plausible explanation may be comorbidity between CMDs and pain, which may lead to limitations in physical performance [45].

Work ability in relation to mental demands was not associated with days of sickness absence, which might seem contradictory. As CMDs may affect cognitive functioning, lead to rumination, and employees´ withdrawal from social contact, work ability in relation to mental demands could potentially lead to prolonged or additional sickness absence [8]. The overall rating of work ability in relation to mental demands was worse than for physical demands (see Table 1). It is possible that the ceiling effect and low variance of this variable made it difficult to establish a statistical relationship.

High job demands have previously been identified as a possible predictor of sickness absence [20]. Our study supports an association between psychological demands and increased sickness absence for employees on sickness absence due to CMDs. High job demands, without the resources to cope with them, may lead to depletion of an employee’s energy, resulting in a health impairment process [46].

Having a high certainty of returning to work within three months was associated with fewer days on sickness absence during the 18-month follow-up, in line with earlier research [19, 20]. This could mirror the attitudes and beliefs of the individual, however, a high certainty of return to work could also be interpreted as reflecting the possibilities of making accommodations at the workplace. Future research could explore employees’ certainty of return to work to understand more about this relationship. Healthcare personnel, who have been identified as key stakeholders in both prevention of sickness absence and facilitating RTW for this group [47], may inquire about expectations of sickness absence when handling patients on sickness absence due to CMDs.

Symptom severity was not included in the final model, contrary to what previous research has found in terms of RTW [19] and sickness absence [48]. A qualitative interview study with participants from the current study sample and their managers highlighted that remaining symptoms may hamper the RTW process and that high psychological demands may further exacerbate these symptoms [49]. Ekberg et al. [50] concluded that factors in the work environment are more influential when it comes to a delayed RTW compared with health-related factors. Interventions to facilitate RTW tend to focus on the individual and do not address issues at the workplace [13]. The need for collaboration in the RTW process has been emphasized by patients on sickness absence and should be considered in intervention development [51]. Our study emphasis the importance of the workplace, also in a primary care setting, and the appraisal of demands, work ability, and return to work expectations.

The results from this study indicate a possible avenue for future research in how employees on sickness absence due to CMDs view their possibilities to return to work. The identification of employees with different needs for support could help prioritize resources. In addition, psychological demands, work ability in relation to physical demands, and certainty of RTW within 3 months, are all potential modifiable factors. These could be considered in future intervention development.

### Strengths

The study has a prospective longitudinal design. The response rate to the baseline questionnaire was high, which minimizes risk of selection bias.

Validated measures to assess symptoms and work-related factors were used; however, the question concerning certainty of return to work within three months has not been validated and possible misclassification may be present. If so, this would be expected to be non-differential and not influenced by other variables in the study. As data on sickness absence was register-based, the internal validity was un-affected by non-responses or recall bias. The follow-up period was 18 months. The response rate was high, which strengthens the results.

### Limitations

Some limitations of the study need to be noted. Firstly, our aim was to analyze the number of sickness absence days during the 18-month follow-up, however, due to methodological considerations and the properties of the data, a GEE analysis was chosen which limits the possibilities of interpreting the data in sickness absence days. The relatively small sample size and exploratory approach with multiple comparisons may introduce a risk of increasing type 1 error. There is a risk that the factors we found may not have been the most important and that our choice of analysis may have excluded other more important factors. Lengthy questionnaires, however, may affect the response rate, which was considered when designing the questionnaire. Possible predictive factors were collected when the person was on sickness absence and not exposed directly to their work environment. We do not know if this affected the validity of the responses to the work-related variables and the results should be interpreted with caution. Including objective work-related measures could have strengthened the analysis of the workplace role. Future research could consider including objective measures for organizational variables, such as turnover or sickness absence rates.

Participants who accepted participation may have had a better relationship with their manager, as manager involvement was an inclusion criterion for participation in the RCT, introducing a risk of selection bias. This could result in an overrepresentation of employees with a good relationship with their manager in the analysis, and a risk of underestimating the association between fair leadership, social support, and sickness absence during the follow-up.

The small sample size restricted subgroup analyses and detection of small effects due to lack of statistical power. Comorbidity between CMDs is common, and the primary diagnosis may not necessarily be the dominant one in terms of symptom burden [52]. Therefore, we did not make separate analysis for baseline diagnosis.

### Generalizability

The findings could be generalized to other employees on sickness absence up to 3 months due to CMDs in Swedish primary healthcare or other countries with similar healthcare and social insurance systems. Further, job demands and the employee’s appraisal and capacity to deal with them should be possible to generalize to other countries as well, even though level of demands or type of demands may differ between sectors.

A high proportion of participants were female, limiting the possibility of generalizing the findings to male employees. However, exposure to factors such as psychological demands at work appears to affect women and men similarly when it comes to health outcomes [53]. A larger proportion of men did not accept participation (26% of the eligible participants were male, compared with 15% among those who accepted participation). This gender composition is similar to other studies; however, we do not know if the male employees included in our study differed from those not accepting participation.

## Conclusions

Psychological demands, work ability in relation to physical demands, and certainty to RTW within 3 months were associated with future sickness absence. Efforts to manage demands in the workplace and the employees’ work ability in relation to demands could be considered when designing interventions with workplace involvement for employees on sickness absence due to CMDs. Ratings of job demands and work ability could serve as basis for a structured inventory to assist healthcare personnel in addressing workplace factors.

## Data Availability

The data is not publicly available due to containing information that could compromise the privacy of the study participants. Reasonable inquiries about access may be sent to Karolinska Institutet, Institute of Environmental Medicine, Unit of Intervention and Implementation Research for Worker Health, Box 210, 171 77 Stockholm or by contacting the Research and Data Office at Karolinska Institutet: rdo@ki.se. The Swedish Ethical Review Authority will then be contacted for permission.

## Abbreviations

CMDs: Common mental disorders
COPSOQ: Copenhagen Psychosocial Questionnaire
DCSQ: Demand-control-support questionnaire
EQ-5D: European quality of life– five dimensions questionnaire
GEE: General estimating equation
GP: General practitioner
HAD: Hospital Anxiety and Depression scale
KSQ: Karolinska sleep questionnaire
OECD: Organisation for Economic Cooperation and Development
PCC: Primary care center
QPSNordic: General Nordic Questionnaire for psychological and social factors at work
RCT: Randomized controlled trial
RTW: Return to work
s-ED: self-rated Exhaustion Disorder
SF-36: Shortform-36
VIF: Variance inflation factor
WAI: Work ability index
QIC: Quality under the information criterion
